# Traditional Chinese Medicine and Colorectal Cancer: Implications for Drug Discovery

**DOI:** 10.3389/fphar.2021.685002

**Published:** 2021-07-01

**Authors:** Qiang Sun, Man He, Meng Zhang, Sha Zeng, Li Chen, Hui Zhao, Han Yang, Maolun Liu, Shan Ren, Haibo Xu

**Affiliations:** State Key Laboratory of Southwestern Chinese Medicine Resources, Department of Pharmacology, School of Pharmacy, Chengdu University of Traditional Chinese Medicine, Chengdu, China

**Keywords:** traditional Chinese medicine, colorectal cancer, drug discovery, bioactive ingredient, effective substance

## Abstract

As an important part of complementary and alternative medicine, traditional Chinese medicine (TCM) has been applied to treat a host of diseases for centuries. Over the years, with the incidence rate of human colorectal cancer (CRC) increasing continuously and the advantage of TCM gradually becoming more prominent, the importance of TCM in both domestic and international fields is also growing with each passing day. However, the unknowability of active ingredients, effective substances, and the underlying mechanisms of TCM against this malignant tumor greatly restricts the translation degree of clinical products and the pace of precision medicine. In this review, based on the characteristics of TCM and the oral administration of most ingredients, we herein provide beneficial information for the clinical utilization of TCM in the prevention and treatment of CRC and retrospect the current preclinical studies on the related active ingredients, as well as put forward the research mode for the discovery of active ingredients and effective substances in TCM, to provide novel insights into the research and development of innovative agents from this conventional medicine for CRC treatment and assist the realization of precision medicine.

## Introduction

Colorectal cancer (CRC) is among the most common types of malignant tumors diagnosed globally worldwide and the second only to the death of lung cancer (Chen et al., 2020; La Vecchia and Sebastian, 2020), which accounts for approximately 10% of all annually diagnosed cancers and nearly 881,000 cancer-related deaths in 2018 (Bray et al., 2018). Unfortunately, the situation is becoming more severe. According to the latest research, there were 940,000 deaths attributed to CRC worldwide in 2020, with the deaths still increasing (Sung et al., 2021). Besides, it is the second most common tumor diagnosed in women and the third in men. It is worth mentioning that the incidence and mortality of women are approximately 25% lower than those in men (Dekker et al., 2019). Since the early symptoms are not extremely obvious, most patients are in the late stage after diagnosis. This cancer not only causes damage to the digestive system but also injures the lymph, liver, lung, bone, and so on, if the tumor metastases. Therefore, searching for an effective treatment schedule has become a research hotspot of scholars nationally and internationally.

The causes of CRC are relatively complicated and are mostly related to genetic, lifestyle, obesity, and environmental factors (Botteri et al., 2008; Kyrgiou et al., 2017; Siegel et al., 2020). According to incomplete statistics, surgical treatment is often used in the treatment of patients with nonmetastatic CRC, and most patients will have the disposition to resect part of the rectum or colon. In clinical practice, the recurrence and metastasis of advanced CRC are often treated with reoperation, chemotherapy, radiotherapy, targeted therapy, or other comprehensive treatments. Although great advances had been made in the diagnosis and treatment of CRC, the prognosis for patients remains unsatisfactory (Salaga et al., 2014). In terms of agent intervention, 5-fluorouracil (5-FU) is currently a significant part of palliative and adjuvant systemic chemotherapy for CRC. Over the past decades, a variety of regulatory strategies such as the implementation of 5-FU-based combination regimens and 5-FU pro-drugs have been developed and tested to enhance the antineoplastic activity and to reduce clinical resistance. However, the response rate of patients to these treatments remains unfavorable, and the efficacy of 5-FU-based therapy is frequently compromised by the development of chemotherapy resistance (Marjaneh et al., 2018a; Vodenkova et al., 2020). In addition, irinotecan hydrochloride is a widely used broad-spectrum cytotoxic agent for patients with advanced CRC. Unfortunately, in recent years, clinical practice has corroborated that there are fatal diarrhea dehydration, gastrointestinal damage, abdominal spasmodic pain, fever, and other adverse reactions (Chamseddine et al., 2019; Cheng et al., 2019; Hattori et al., 2019). Thus, more efficacious treatment strategies and approaches for medical intervention, especially the agent with high efficacy and few side effects, are an unmet medical need.

Traditional Chinese medicine (TCM), a predominant source of natural medicines and herbal products, are essential sources for exploiting anti-CRC agents (Kong et al., 2020). At present, although there is no convincing evidence in large-scale randomized controlled trials (RCTs) to support the therapeutic effect of TCM, a recent survey indicates that 20–30% of patients in Indonesia tend to use TCM to treat various diseases (Pengpid and Peltzer, 2018). Recent studies have found that TCM can be used as an effective auxiliary method to reduce the incidence rate of CRC (Ye et al., 2015; Wang et al., 2020). The effective components in Chinese herbs can destroy the living environment of cancer cells, promote apoptosis, enhance the individual’s immunity, and eliminate the pathogens through the autoimmune system, so as to achieve the anticancer effect (Yan et al., 2017b; Zhang et al., 2020d; Qiao et al., 2020). Besides, TCM can be combined with other chemotherapy agents to reduce the adverse reactions caused by chemotherapy and significantly improve the quality of life of patients. Consequently, prior to the development of conclusive evidence-based available pharmacological therapies, the clinical utilization of TCM proves to be a nonignorable strategy for treating various diseases including CRC. The expanding knowledge of TCM in benefiting the treatment of CRC, against the extremely long period of modern drug discovery, has impelled researchers to excavate potential efficacious and secure therapies by the application of TCM, which is considered as “a natural combinatorial chemical gift library” drawing advantage from ancient practical experiences. Following the abovementioned thoughts, in this review, we first gave a brief introduction about the TCM products that have been approved for clinical research. Then, we summarized the scientific literature on the prevention and treatment of CRC with TCM and its bioactive compounds and reviewed the main molecular mechanisms involved in these processes. Finally, based on the characteristics of TCM, we put forward a slice of thoughts and prospects in future research, including reverse pharmacology guiding preclinical research, high-throughput screening of anti-CRC active ingredients, nanotargeted enrichment strategy, and gut-microbiota-mediated effect. This review will update the understanding of the effective material basis of anti-CRC with TCM and guide the discovery of novel agents from TCM.

## Antitumor Status of Traditional Chinese Medicine and Its Clinical Trials on Colorectal Cancer

### Antitumor Status of Traditional Chinese Medicine

As a long-standing science and culture, TCM has a centuries-old history of clinical use in the treatment of considerable maladies for thousands of years, and it has made contributions to the prosperity and civilization of the Chinese nation and the surrounding countries. Unlike chemical or biological products, TCM is usually applied as a prescription to achieve the treatment goal of related diseases. Consequently, in clinical practice, TCM formulas are prescribed by doctors under the guidance of ancient empirical philosophies such as Yin-Yang, monarch (*Jun*), minister (*Chen*), assistant (*Zuo*), and guide (*Shi*) (Dong et al., 2018). In recent years, with the acceleration of modernization and internationalization of TCM, the application of TCM in cancer treatment has become increasingly prominent. For instance, several reviews have described the wide application of TCM therapy on cancer treatment (Wang et al., 2018; Xiang et al., 2019; Liu et al., 2020c). Different from the idea of Western medicine, TCM emphasizes more on “holistic concept” and “survival with tumor,” whose treatment goal is not only to kill cancer cells and reduce the size of the tumor but also to improve the quality of life and prolong the survival span of patients (Han et al., 2016). In addition to underlining the holistic health balance, TCM itself at least provides precious sources of anticancer agents, lead compounds, or adjuvant complements for novel drug discovery. TCM is rich in a battery of effective components, which carries the coordinated regulation of multitarget and multieffect. In the field of drug discovery, successful discovering of exhilarating compounds derived from TCM such as camptothecin, paclitaxel, and curcumin has brought much confidence for scientists to excavate natural antitumor agents (Hesari et al., 2019; Qu et al., 2019; Narayan et al., 2020).

### Clinical Trials of Traditional Chinese Medicine on Colorectal Cancer

The ultimate goal of theoretical research is to successfully apply secure and effective agents to clinical practice. In this era of “precision medicine” and “evidence-based medicine,” it is extremely paramount to carry out large-scale randomized controlled trials of TCM to conclusively confirm whether it is effective in the treatment of related diseases. In order to promote the modernization and internationalization of TCM, the Chinese government launched a grand plan to expand the basic and clinical research of TCM in 2007, which has achieved great results (Qiu, 2007). By setting “colorectal cancer” or “CRC” as the keywords, we searched the website of ClinicalTrails.gov (*https://clinicaltrials.gov/*) and found that there were 5,833 kinds of clinical studies that have been conducted with CRC (on January 28, 2021), of which 38 were related to TCM (in addition to some dietary supplements from botany), including Chinese medicine formula (CMF), Chinese herbal extracts, and Chinese herbal single compounds (Table 1). These clinical projects have brought powerful confidence for the anti-CRC of the active ingredients in TCM, which will promote and accelerate the clinical transformation of TCM products against CRC.

**TABLE 1:** Clinical trials for TCM in treating CRC registered at ClinicalTrials.gov.

Study title	Drugs involved	Status	Identifier
Strengthening the spleen and reducing phlegm Method in improving radical resection rate of colorectal cancer	Jianpi Huatan dispensing granule	Not recruiting, N/A	NCT03716063
Huaier granule as adjuvant therapy for colorectal cancer after radical surgery	Huaier granule	Unknown, N/A	NCT02796820
Simo decoction and acupuncture on POI in colorectal cancer	Simo decoction	Completed, phase 3	NCT02813278
Adjuvant chemotherapy combined with Huaier granule for treating high-risk stage II and stage III colorectal cancer	Huaier granule	Unknown, N/A	NCT02785146
Fuzheng Yiliu-1010	Fuzheng Yiliu formulation	Recruiting, phase 2	NCT04459754
Study of TCM syndrome of hepatocellular carcinoma and colorectal cancer based on system science	Bushen-Jianpi dedoction/Cinobufotalin injection	Recruiting, phase 1	NCT03189992
Pomegranate extract supplementation in colorectal cancer patients	Pomegranate extract	Completed, phase 1/2	NCT01916239
Effect of annona muricata leaves on colorectal cancer patients and colorectal cancer cells	Annona muricata extract	Completed, phase 1	NCT02439580
The efficacy of silymarin as adjuvant therapy on colorectal cancer patients undergoing FOLFIRI treatment	Silymarin	Completed, phase 4	NCT03130634
Does dietary nitrate supplementation improve aerobic performance	Beetroot juice	Not recruiting, phase 4	NCT02319356
Preventive strategies in colorectal carcinogenesis production and meat processing	Pomegranate extract	Completed, N/A	NCT02473302
Gemcitabine combined with mistletoe in treating patients with advanced solid tumors	Mistletoe extract	Terminated, phase 1	NCT00049608
Genistein in the treatment of metastatic colorectal cancer	Genistein	Completed, phase 1/2	NCT01985763
Efficacy of ginseng for patients on regorafenib	Ginseng	Terminated (funder terminated)	NCT02581059
Safety and effectiveness study of preoperative artesunate in stage II/III colorectal cancer (NeoART-V)	Artesunate	Recruiting, phase 2	NCT03093129
Avastin/FOLFIRI in combination with curcumin in colorectal cancer patients with unresectable metastasis	Curcumin	Completed, phase 2	NCT02439385
A pilot study of PPX in women with metastatic colorectal cancer	Paclitaxel Poliglumex	Completed, phase 1	NCT00598247
A safety and effectiveness study of preoperative artesunate in stage II/III colorectal cancer	Artesunate	Recruiting, phase 2	NCT02633098
Study of andrographolides with or without capecitabine to treat colorectal cancer	Andrographolides	Terminated (low accrual rate)	NCT01993472
Effect of curcumin on dose-limiting toxicity and pharmacokinetics of irinotecan in patients with solid tumors	Curcumin	Completed, phase 1	NCT01859858
AZD2171 + chemotherapy in advanced NSCLC, colorectal cancer, or other cancers suitable for treatment with capecitabine (non-small-cell lung cancer patients closed to enrollment as 8/9/07)	Paclitaxel	Completed, phase 1	NCT00107250
Panitumumab skin toxicity prevention trial	Lycopene	Recruiting, phase 2	NCT03167268
Sulindac and plant compounds in preventing colon cancer	Curcumin/rutin/quercetin	Completed, N/A	NCT00003365
Curcumin for the prevention of colon cancer	Curcumin	Completed, phase 1	NCT00027495
Combining curcumin with FOLFOX chemotherapy in patients with inoperable colorectal cancer	Curcumin	Completed, phase 1/2	NCT01490996
Resveratrol in treating patients with colorectal cancer that can be removed by surgery	Resveratrol	Completed, phase 1	NCT00433576
Cancer-associated thrombosis and isoquercetin (CATIQ)	Isoquercetin	Not recruiting	NCT02195232
Preventive effect of enoxaparin, pentoxifylline, and ursodeoxycholic acid to radiation-induced liver toxicity	Ursodeoxycholic acid	Completed, phase 2	NCT01149304
Berberine chloride in preventing colorectal cancer in patients with ulcerative colitis in remission	Berberine chloride	Not recruiting	NCT02365480
Paclitaxel and bortezomib in treating patients with metastatic or unresectable malignant solid tumors	Paclitaxel	Completed, phase 1	NCT00667641
Curcumin in combination with 5FU for colon cancer	Curcumin	Not recruiting	NCT02724202
Study investigating the ability of plant exosomes to deliver curcumin to normal and colon cancer tissue	Curcumin	Not recruiting	NCT01294072
Resveratrol for patients with colon cancer	Resveratrol	Completed, phase 1	NCT00256334
Phase III trial of gemcitabine, curcumin, and celebrex in patients with metastatic colon cancer	Curcumin	Unknown	NCT00295035
Radiation therapy and capecitabine with or without curcumin before surgery in treating patients with rectal cancer	Curcumin	Not recruiting	NCT00745134
Efficacy and safety evaluation of traditional Chinese medicine in the treatment of advanced colorectal cancer	Unknown	Not recruiting, N/A	NCT02923622
Dietary bioflavonoid supplementation for the prevention of neoplasia recurrence	Flavonoids	Suspended, phase 2	NCT00609310

### Traditional Chinese Medicine Preclinical Trials on Colorectal Cancer

At present, due to the overall regulatory effect of TCM on human body and low toxicity and side effects, the research on diseases including CRC is increasing all over the world (Fan et al., 2020). At the same time, great progress has been made in its pharmacological research and clinical application. Also, the high R&D cost and long R&D time of chemosynthetic agents also prompt pharmaceutical scientists to enhance their efforts in mining novel and effective antitumor agents from TCM ingredients. In general, rapid advancement in the discovery of active components of TCM has provided an array of opportunities for developing novel anti-CRC therapeutic strategies.

### Anti-Colorectal-Cancer Effect of Chinese Medicine Formula

As an ancient science and culture, Chinese medicine formula (CMF) has a centuries-old history in clinical treatment of considerable ailments for thousands of years, and TCM has made many contributions to the prosperity and civilization of China and other neighboring countries (Luan et al., 2020; You et al., 2020). The rational compatibility of TCM should follow the principle of “monarch (*Jun*), minister (*Chen*), assistant (*Zuo*), and guide (*Shi*)” in the theory of TCM, rather than the simple combination of various herbal medicine or compounds. In this theory, “monarch (*Jun*), minister (*Chen*), assistant (*Zuo*), and guide (*Shi*)” represent the corresponding agents in the prescription, respectively. “Monarch drug” plays a major therapeutic role and “minister drug” can enhance the curative effect of “monarch drug,” while “assistant drug” can cooperate with “monarch drug” and “minister drug” to undermine possible side effects. “Guide drug” can lead other components to the pathogenic part of the ingredients. The CMF is rich in an ocean of components and has complex pharmacological effects, which can exert an antitumor effect through a variety of approaches (Liu et al., 2015; Wan et al., 2018; Lee et al., 2020). It has been verified that a variety of CMF can significantly improve the quality of life and survival rate of CRC patients through the overall regulatory effect (Chu et al., 2015; Lin et al., 2017). For example, Shaoyao decoction (SYD), a traditional CMF formulated by the master in Jin-Yuan Dynasty Liu Wan-Su, consisting of Shaoyao, Binglang, Dahuang, Huangqin, Huanglian, Danggui, Guangui, Gancao, and Muxiang, has been proved to significantly elevate the survival rate of the mice and reduced the incidence of colonic neoplasms (Lin et al., 2014). Additionally, it is reported that herbal formula Huang Qin Ge Gen Tang can potentiate the antitumor activity of 5-FU by regulating the E2F1/TS pathway (Liu et al., 2018).

When it comes to CMF, we have to call to mind the Chinese medicine pair in TCM. The Chinese medicine pair is a part between compound CMF and single Chinese medicine. It is a combination of two commonly used herbs in clinical practice of TCM, and it is also a basic form of compatibility and application in TCM. Generally speaking, the Chinese medicine pair possesses a better curative effect or lower toxicity than single Chinese medicine, which is the accumulation of ancient empiricism philosophy (Zhou et al., 2017; Xu et al., 2020). With the method of network pharmacology, researchers found that the herb pair “Huang Lian-Gan Jiang” can regulate 500 biological processes and 70 molecular functions, affect 62 related signaling pathways, and then, exert the prevention and treatment of CRC (Gong et al., 2019). In a study on the herb pair *Hedyotis diffusa* and *Sculellaria barbata* against CRC, it was found that this compatibility of medicines inhibited the tumor growth both *in vitro* and *in vivo*, which might be related to apoptosis induction through the EGFR/PPAR gamma/PI3K/AKT pathway (Lu et al., 2020a). Another study showed that the combination pair *Panax ginseng* and *Veratrum nigrum* decreased cell proliferation *via* inducing cell cycle arrest and apoptosis of CT 26 and HT 29 cells, as well as suppressed metastatic abilities of the abovementioned two cells including epithelial–mesenchymal transition (EMT), migration, and invasion (Kee et al., 2018).

With the development of TCM pharmaceutics, some CMFs have been developed into Chinese patent medicine for the prevention and treatment of CRC. Chinese patent medicine is the novel CMF developed by the modern pharmaceutical technology under the TCM principle of syndrome differentiation and treatment, which can exert an anti-CRC effect by interfering with multiple processes. For instance, as the report goes, Da Huang Zhe Chong Pill halted the CRC liver metastasis by ameliorating exosomal CCL2 primed premetastatic niche (Chen et al., 2019). Pian Zi Huang, a prescription preparation, can suppress the proliferation and induce apoptosis of CRC stem cells by inhibiting the Notch1 signaling pathway (Wei et al., 2014; Qi et al., 2016). Additionally, it can effectively surmount MDR of 5-FU and block the EMT in human colorectal carcinoma cells by inhibiting the TGF-beta signaling pathway (Shen et al., 2014). More detailed information concerning anti-CRC of CMF is depicted in Table 2.

**TABLE 2:** CMFs for anti-CRC and corresponding mechanisms.

CMF name	Composition	Cell lines/model	Dose	Detail	Mechanism	Ref
Fu Fang Yi Liu Yin formula	Astragali Radix, Ganoderma lucidum, semen armeniacae amarum, *H. diffusa* Willd., Aconiti Lateralis Radix Praeparata, *Glycyrrhiza glabra* Linn., Radix Panacis Quinquefolii, and Platycodi Radix	HCT 116 cells	3–15 mg/ml	*In vitro*	Inhibit cell proliferation and induce apoptosis and block cell at G_0_/G_1_ phase. *In vivo*, inhibit tumor growth	Dong et al. (2020)
		SW 480 cells	3–15 mg/ml	*In vitro*		
		BALB/c mice	2.4 mg/g	*In vivo*		
Yi Fu Zi Bai Jiang San	Semen coicis, monkshood, and Herba Patriniae	HCT 116 cells	15.625–62.5 μg/ml	*In vitro*	Block tumor initiation and progression, increase immune function, regulate gut flora, alter cell growth, and reduce phosphorylation of β-catenin	Sui et al. (2020)
		MC 38 cells	15.625–62.5 μg/ml	*In vitro*		
		C57BL/6 J mice	3.825–15.3 g/kg	*In vivo*		
*Astragalus atractylodes* mixture	*Astragalus membranaceus*, *Atractylodes macrocephala*, *Actinidia arguta*, *Curcuma aromatica*, *Benincasa hispida*, and *Ficus pumila*.	HCT 116 cells	0.5–16 mg/ml	*In vitro*	Inhibit hypoxia-induced ROS generation, migration and VM formation, as well as HIF-1 alpha and MMP2 expression	Zong et al. (2020)
		LoVo cells	0.5–16 mg/ml	*In vitro*		
Xiang Sha Liu Jun Zi decoction	Radix Codonopsis, rhizoma *Atractylodis macrocephalae*, radix glycyrrhizae, *Poria*, Pericarpium citri reticulatae, *Pinellia* tuber, Radix Aucklandiae, and Fructus Amomi	Patients with stage III or IV CRC	Unknown	*In vivo*	Unknown	Hong et al. (2020)
Wu Mei Wan	Fructus Mume, rhizoma coptidis, Herba Asari Mandshurici, Ramulus Cinnamomi, Radix Ginseng, Radix Aconiti Lateralis Preparata, Pericarpium Zanthoxyli Bungeani, Rhizoma Zingiberis, Cortex Phellodendri Amurensis, and Radix Angelicae Sinensis	C57BL/6 J mice	5.8 g/kg	*In vivo*	Improve the survival rate and attenuate symptoms, reduce proliferation of tumor cells, decrease the expression of p65, IL-6, and p-STAT3, decrease *Bacteroidetes*, and increase *Firmicutes*	Jiang et al. (2020)
Zuo Jin Wan	*Coptis chinensis* Franch. and Evodia ruticarpa	HCT 116 cells	100–300 µM	*In vitro*	Induce apoptosis through the PI3K-Akt signaling pathway	Huang et al. (2020b)
		HT 29 cells	100–300 µM	*In vitro*		
Zuo Jin Wan	*Coptis chinensis* Franch. and Evodia ruticarpa	SW 403 cell	25–800 μg/ml	*In vitro*	Increase G_1_ arrest in cell cycle, induce apoptosis, suppress cell migration and invasion, and decrease the expression of 5-HTR1D and β-catenin	Pan et al. (2017)
Compound sophorae decoction	*Sophora flavescens* and *Sanguisorba officinalis*, *Indigo naturalis*, *Bletilla striata*, *Panax notoginseng*, and *Glycyrrhiza uralensis*	C57BL/6 J mice	0.1614 g	*In vivo*	Execute UCRCC-inhibitory activity by counteracting inflammatory responses and rescuing detuning of apoptosis as well as neutralizing overactive mitophagy	Deng et al. (2019)
Qing Jie Fu Zheng granules	*Scutellaria barbata*, malt, *Hedyotis diffusa*, and *Astragalus mongholicus*	HCT 8 cells	0.5–2 mg/ml	*In vitro*	Inhibit proliferation and induce apoptosis by suppressing the PI3K/AKT and ERK pathways	Yang et al. (2019a)
		HCT 116 cells	0.5–2 mg/ml	*In vitro*		
Si Jun Zi decoction	*Codonopsis pilosula*, *Poria cocos*, *Atractylodes macrocephala*, and radix liquiritiae	Balb/c mice	45 g/kg	*In vivo*	Increase survival rate and reduce liver metastasis, elevate plasma GM-CSF level, and increase the number of macrophages but not neutrophils in the spleen	Zhou et al. (2019a)
Chang Wei Qing	*Astragalus membranaceus*, *Atractylodes macrocephala*, *Codonopsis pilosula*, *Akebia quinata*, *Polyporus umbellatus*, *Coix* seed, *Vitis quinquangularis* Rehder, and *Sargentodoxa cuneata*	C57BL/6 J mice	5, 10 mg/kg	*In vivo*	Restore colon length, decrease tumor number and size, reduce colitis score, suppress expansion of *F. prausnitzii* population, and inhibit activity of beta-glucuronidase and leakage of d-lactose and endotoxin	Wan et al. (2019)
Su Yang decoction	Broccoli and green cabbage	HT 29 cells	10–200 μg/ml	*In vitro*	Inhibit colon cancer cell proliferation and induce G_1_ phase arrest and induce the cleavage of poly (ADP-ribose) polymerase, tumor necrosis factor superfamily member 10, X-linked inhibitor of apoptosis	Ge et al. (2019)
		LS 174-T cells	10–200 μg/ml	*In vitro*		
		CRL-1790 cells	10–200 μg/ml	*In vitro*		
Jian Pi Jie Du decoction	*Astragalus membranaceusceus*, *Panax quinquefolius, Atractylodes macrocephala*, *Poria cocos*, *Coix* seed, *Smilax china*, *Hedyotis diffusa, Sculellaria barbata, Paris polyphylla, Actinidia argut, and gGlycyrrhiza uralensis* Fisch.	HCT116 cells	0.3125–2.5 mg/ml	*In vitro*	Inhibit viability and proliferation, induce apoptosis, suppress migration, invasion, and angiogenesis by inhibiting the mTOR/HIF-1α/VEGF signaling pathway, decrease the CD34 and VEGF, and downregulate the mTOR/HIF-1α/VEGF pathway	Peng et al. (2018)
		HT29 cells	0.3125–2.5 mg/ml	*In vitro*		
		LoVo cells	0.3125–2.5 mg/	*In vitro*		
		SW48 cells	0.3125–2.5 mg/ml	*In vitro*		
Tian Xian liquid	Radix Ginseng, *Cordyceps*, Radix Astragali, Radix Glycyrrhizae, rhizoma, margarita, Fructus lycii, *Ganoderma lucidum*, Fructus ligustri lucidi, and Herba Scutellariae barbatae	HT29 cells	0.625–5% (v/v	*In vitro*	Inhibit proliferation, upregulate the p21 mRNA and protein, downregulate G_1_ phase cell cycle protein, cyclin D1 mRNA and protein, and reverse multidrug resistance	Leigh et al. (2017)
		Nude mice	200 µl	*In vivo*		
Yi Ai Fang	*Astragalus membranaceus*, *Atractylis ovate*, *Actinidia arguta, Curcuma zedoaria,* and *Benincasa hispida*	BABL/c mice HCT 116 cells	8–32 mg/kg	*In vivo*	Restrain the formation of vasculogenic mimicry through the HIF-1α/EMT pathway, inhibit growth of the xenografted tumors, enhance expression of E-cd and claudin-4, and decrease the expression of HIF-1α and VIM	Hou et al. (2016)
			25–200 μg/ml	*In vitro*		
Huang Qin decoction	*Scutellaria baicalensis* Georgi., *Paeonia lactiflora* Pall., *Glycyrrhiza uralensis* Fisch., and *Ziziphus jujuba* Mill.	C57BL/6 mice	9.1 g/kg	*In vivo*	Inhibit AOM/DSS-induced CRC and the production of inflammatory cytokines and increase antioxidant capacity both in chronic DSS- and AOM/DSS-treated mice	Chen et al. (2016)
Shen Ling Bai Zhu San	Radix et rRhizoma gGinseng, *Poria*, Rhizoma Atractylodis Macrocephalae, semen Lablab Album, Rhizoma Dioscoreae, Radix et Rhizoma Glycyrrhizae, *Plumula nelumbinis*, Fructus Amomi, semen coicis, and Radix Platycodonis	C57BL/6 J mice	3.64–14.56 g/kg	*In vivo*	Supress colitis-associated CRC through the inhibition of EMT and myeloid-derived suppressor infiltration	Lin et al. (2015)
		SW480 cells	6–16 mg/ml	*In vitro*		
		HCT116 cells	6–16 mg/ml	*In vitro*		
Jian Pi Hua Yu decoction	*Atractylodes macrocephala* Koidz., *Euphorbia humifusa* Willd., *Salvia miltiorrhiza* Bunge., *Paris polyphylla* Sm., *Curcuma phaeocaulis* Val., *Scutellaria barbata* D. Don., and *Artemisia capillaris* Thunb.	SW480 cells	0.25–8 mg/ml	*In vitro*	Decrease viability, induce G_0_/g_1_-phase cell cycle arrest and induce apoptosis, enhance the expression of p27, cleaved PARP, cleaved caspase-3, and bax, and decrease the levels of PARP, caspase-3, Bcl-2, CDK2, CDK4, CDK6, cyclin D1, cyclin D2, cyclin D3, and cyclin E1	Xi et al. (2015)
Yi Qi Fu Sheng formula	*Codonopsis pilosula*, *Atractylodes macrocephala, Poria cocos*, Radix liquiritiae, *Myristica fragrans*, and Fructus Akebiae	HCT-116 cells	50–250 mg/ml 200–800 mg/kg	*In vitro*	Inhibit migration/invasion of CRC by inhibiting the activation of ERK/MAPK signaling pathways	Deng et al. (2013b)
		Athymic mice		*In vivo*		
Jian Pi Jie Du recipe	Radix Astragal, Rhizoma *Atractylodis macrocephala*, wild grapevines, Fructus Akebia, *Salvia chinensis* Benth., and *Evodia rutaecarpa*	LoVo cells	12.5–400 μg/ml	*In vitro*	Inhibit invasive and migratory and reduce the transcriptional activities of EMT-associated factors snail and E-cadherin. *In vivo*, inhibit liver and lung metastasis of orthotopic CRC, prolonging the survival time	Liu et al. (2017b)
		Nude mice	250–1,000 mg/kg	*In vivo*		
Huang Lian Jie Du decoction	*Coptis chinensis* Franch., *Phellodendron amurense* Rupr., *Gardenia jasminoides* J. Ellis, and *Scutellaria baicalensis* Georgi.	Athymic mice	50–200 mg/kg	*In vivo*	Promote renewal of the intestinal cell wall, induce presentation of CD44-postive cells, initiate the expression of stemness-associated genes, elevate transcriptional products of the downstream Wnt signaling of CD44, and reduce diarrhea and intestinal damage	Chan et al. (2020)
Xiao Ai Jie Du decoction	*Hedyotis diffusa* and *Codonopsis pilosa, Sophora flavescens*, and *Zingiber officinale*	Patients who fulfill the criteria	Unknown	*In vivo*	Unknown	Zhou et al. (2019c)
BP10A	*Descurainiae sophia* semen and *Peucedani praeruptorum* radix	HCT-116 cells KM12SM cells	6.25–25 μg/ml 25–200 μg/ml	*In vitro*	Delay tumor growth and enhance the antitumor activity of each anticancer drug and delay tumor growth	Kim et al. (2019b)
				*In vitro*		
Ge Gen Qin Lian decoction	Radix Puerariae, *Scutellariae* radix, Coptidis Rhizoma, and liquorice	BALB/c mice	300–7500 mg/kg	*In vivo*	Enrich related intestinal microorganisms, increase the proportion of CD8^+^ T cells in peripheral blood and tumor tissues, increase the expression of IFN-γ, downregulate PD-1, and increase IL-2 levels	Lv et al. (2019)
Zhi Zhen Fang formula	Radix Astragali, fFructus ligustri lucidi, semen coicis, *Salvia chinensis*, *Vitis quinquangularis* Rehd., *Actinidia arguta*, and *Cyperus rotundus* L.	HCT-116 cells HCT-8 cells	25–1,600 μg/ml	*In vitro*	Enhance the sensitivity of chemotherapeutic drugs and induce apoptosis, inhibit the hedgehog pathway, inhibit tumor growth, and reduce Gli1 levels	Sui et al. (2017)
		Athymic mice	25–1,600 μg/ml	*In vitro*		
			13.27–53.08 g/kg	*In vivo*		
Teng Long Bu Zhong Tang	*Actinidia chinensis, Solanum nigrum, Duchesnea indica, Atractylodes macrocephala* Koidz., *Poria cocos*, *Coix* seed, mistletoe, and *Scutellaria barbata*	BALB/c mice	22.5, 30 mg/kg	*In vivo*	Inhibit cancer cell growth, elicite apoptosis, and downregulate XIAP and survivin, induce cell senescence, and enhance anticancer effects of 5-Fu	Deng et al. (2013a)
Wei Chang An	*Pseudostellaria heterophylla* Pax., *Atractylodes macrocephala* Koidz., *Poria cocos* Wolf., *Glycyrrhiza uralensis* Fisch., *Sargentodoxa cuneata*, and *Prunella vulgaris* L.	HCT-116 cells	3–9%	*In vitro*	Reduce the rate of metastasis, decrease the expression of β-catenin and MMP-7, and reduce nuclear translocation of β-catenin	Tao et al. (2015)

### Anti-Colorectal-Cancer Effect of Traditional Chinese Medicine Extract

TCM extract is a kind of TCM product with a relatively clear pharmacodynamic material basis and strict quality standard, which is a novel product form in the international natural medicine market and can be widely used in natural health products (Shen et al., 2017; Bu et al., 2020). At present, a variety of preclinical works on the anti-CRC effect of TCM extracts (water extract, ethanolic extract, chloroform extract, etc) have been reported. For example, *Galla chinensis*, a commonly used herbal medicine in East Asia, has been found to inhibit lung metastasis by inducing AMPK-mediated apoptosis and inhibiting the metastasis of CRC cells (Mun et al., 2019). Treatment of HCT116 cells, HT29 cells, SW480 cells, Caco-2 cells, and Colo205 cells with various concentrations of *Antrodia cinnamomea* extract resulted in a dose- and time-dependent decrease in cell viability, indicating that it may induce autophagic cell death *via* the CHOP/TRB3/Akt/mTOR pathway. In addition, in a nude mice model of metastatic CRC cancer, similar experimental results are also observed (Tsai et al., 2018). Another study demonstrated that the *Ampelopsis* ethanolic extract can suppress STAT3 and Src phosphorylation, inhibit STAT3 nuclear localization, and downregulate the expression of STAT3 target genes Mcl-1, Bcl-xL, and MMP-2 in HCT-116 and SW480 cells. These data provide a relevant scientific research basis for the traditional use of *Ampelopsis radix* extract for CRC and a pharmacological clue for the development of a modern anti-CRC agent (Su et al., 2017). *Hedyotis diffusa*, a typical Chinese herb for clearing heat and detoxification, has demonstrated that its chloroform extract can inhibit the activity of human CRC cells by inhibiting the Akt and ERK signaling pathway (Yan et al., 2017a). In SW480 and SW620 cells, *Ginkgo biloba* extract dose-dependently inhibited cell migration and invasion, induced upregulation of LncRNA-p21 expression, and suppressed the expression of extracellular matrix protein fibronectin (Liu et al., 2017a). Overall, the abovementioned documents suggest the TCM extract as a promising therapeutic agent for CRC in clinical settings. More detailed information concerning anti-CRC of TCM extracts is depicted in Table 3.

**TABLE 3:** Anti-CRC effect and mechanism of TCM extract.

Extract type	Source	Cell lines/model	Dose	Detail	Mechanism	Ref
Aqueous extract	Galla Rhois	HT 29 cells	20–100 μg/ml	*In vitro*	Inhibit lung metastasis by inducing AMPK-mediated apoptosis and suppressing metastatic properties of colorectal cancer cells	Mun et al. (2019)
		CT 26 cells	20–100 μg/ml	*In vitro*		
		BALB/c mice	200, 500 mg/kg	*In vivo*		
—	*Ginkgo biloba*	SW 480 cells	200,500 mg/ml	*In vitro*	Inhibit migration and invasion, induce upregulation of LncRNA-p21 expression, and inhibit the expression of extracellular matrix protein fibronectin	Liu et al. (2017a)
		SW 620 cells	200,500 mg/ml	*In vitro*		
—	Medicinal mushroom	HCT 116 cells	1.332–13.32 μg/ml	*In vitro*	Inhibit cell proliferation and promote cell apoptosis, inhibit tumor growth, and inhibit VEGF and MMP-2 and MMP-9 modulation	Jakopovic et al. (2020)
		SW 620 cells	1.332–13.32 μg/ml	*In vitro*		
		BALB/c mice	400, 1,200 mg/kg	*In vivo*		
—	*Pogostemon cablin*	HT 29 cells	5.83–93.2 μg/ml	*In vitro*	Decrease viability, inhibit proliferation and induce cell cycle arrest at the G_0_/G_1_ phase and apoptosis, and suppress growth of CRC	Chien et al. (2020)
		CT 26 cells	5.83–93.2 μg/ml	*In vitro*		
		BALB/c mice	200 mg/kg	*In vivo*		
—	*Scutellaria barbata*	Nude mice	615,1230 mg/kg	*In vivo*	Regulate the expressions of related proteins E-cadherin, Tspan 8 and CXCR4, and Src kinase and reduce orthotopic tumor burden	Yue et al. (2020)
—	*Cudrania tricuspidata* leaf	C57BL/6 J mice	1.5 g/kg	*In vivo*	Reduce the risk of colitis-associated colon cancer *via* the regulation of inflammation, carcinogenesis, and compositional change of gut microbiota	Oh et al. (2020)
—	*Solanum nigrum* leaf	HT 29 cell	0.05–5 mg/ml	*In vitro*	Induce autophagy *via* microtubule-associated protein 1 light chain 3 A/B II accumulation and enhance cytotoxicity in tumor cells	Tai et al. (2013)
		DLD-1 cells	0.05–5 mg/ml	*In vitro*		
—	Pulsatillae Radix	SW 480 cells	5–20 μg/ml	*In vitro*	Inhibit invasion and migration and block the S phase in the cell cycle	Zhang et al. (2019b)
Ethanol extract	*Antrodia cinnamomea*	HCT 116 cells HT 29 cells	50–200 μg/ml	*In vitro*	Upregulate expression of the endoplasmic reticulum stress marker CHOP and its downstream gene TRB3 and induce autophagic cell death and dephosphorylation of Akt and mTOR.	Tsai et al. (2018)
		SW 480 cells Caco-2 cells Colo 205 cells	50–200 μg/ml	In vitroIn vitro		
			50–200 μg/ml	*In vitro*		
			50–200 μg/ml	*In vitro*		
			50–200 μg/ml			
		Nude mice	100–400 mg/kg	*In vivo*		
—	Ampelopsis radix	HCT 116 cells SW 480 cells	50–600 μg/ml	*In vitro*	Suppress STAT3 and Src phosphorylation, inhibit STAT3 nuclear localization, and downregulate the expression of STAT3 target genes Mcl-1, Bcl-xL, and MMP-2	Su et al. (2017)
			50–600 μg/ml	*In vitro*		
—	*Hedyotis diffusa* Willd.	HT 29 cells	0.5–2 mg/ml	*In vitro*	Downregulate the expression of leucine-rich repeat-containing G-protein-coupled receptor 5 and decrease the proportion of SP, inhibit viability and sphere formation, induce cell morphological changes, and suppress messenger RNA expression of several critical genes	Sun et al. (2016)
—	*Hedyotis diffusa* Willd.	BABL/c mice	6 g/kg	*In vivo*	Reduce tumor volume and weight, suppress STAT3 phosphorylation, alter expression pattern of target genes, and decrease cyclin D1, CDK4, and Bcl-2	Cai et al. (2012)
Methanolic extract	*Emilia sonchifolia*	HCT 116 cells	25–100 μg/ml	*In vitro*	Inhibit cell growth, induce apoptosis, promote the mitochondria-dependent and death-receptor-associated protein levels, increase ROS production, and upregulate ATM, p53, and Fas	Lan et al. (2012)
		HT 29 cells	25–100 μg/ml	*In vitro*		
		SW 480 cells	25–100 μg/ml	*In vitro*		
—	*Artemisia absinthium*	HCT 116 cells	100–1,000 μg/ml	*In vitro*	Reduce viability, increase the mRNA and protein levels of Bax, decrease BCL-2, prompt cell cycle arrest, and induce apoptosis by activating the mitochondrial pathway	Nazeri et al. (2020)
—	*Muntingia calabura* L.	Wistar rats	100, 200 mg/kg	*In vivo*	Cause antioxidant enzymic levels to retain near to its normal range and reduce severity of colorectal cancer	Jisha et al. (2020)
—	Immature fruit of *Poncirus trifoliata*	CT-26 cells HCT-116 cells DLD-1 cells	1–20 µM	*In vitro*	Inhibit proliferation and induce autophagy and apoptosis by protein kinase B/mammalian target of rapamycin and 5′-AMP-activated protein kinase pathways	Kim et al. (2020)
			1–20 µM	*In vitro*		
			1–20 µM	*In vitro*		
Chloroform extract	*Hedyotis diffusa* Willd.	SW 620 cells	150–500 μg/ml	*In vitro*	Inhibit proliferation and promote apoptosis, downregulate the survivin, proliferating cell nuclear antigen, cyclin-dependent kinase 4, and Bcl-2, and upregulate Bcl-2-associated X protein	Jakopovic et al. (2020)
		HT 29 cells	150–500 μg/ml	*In vitro*		
		HCT 116 cells	150–500 μg/ml	*In vitro*		
		HCT 8 cells	150–500 μg/ml	*In vitro*		
—	*Scutellaria barbata* D. Don.	HCT 8 cells	50–300 μg/ml	*In vitro*	Inhibit proliferation and promote apoptosis, increase miR-34a expression, and decrease Bcl-2, Notch1/2, and Jagged1 expression	Zhang et al. (2017a)
Ethyl acetate extract	*Selaginella doederleinii* Hieron.	HT 29 cells	10–200 μg/ml 12.5–200 μg/ml	*In vitro*	Inhibit proliferation and induce cell morphological changes, cell cycle arrest, autophagy, and apoptosis, induce loss of mitochondrial membrane potential, increase the autophagic flux, raise the ratio of Bax/Bcl-2, activate caspases, and inhibit growth of xenograft tumors	Li et al. (2020)
		HCT 116 cells SW 620 cells	12.5–200 μg/ml	*In vitro*		
		SW 480 cells	10–200 μg/ml	*In vitro*		
		SW 1116 cells	12.5–200 μg/ml	*In vitro*		
		Nude mice		*In vitro*		
			100–300 mg/kg	*In vivo*		
Hydrophilc extract of manna	*Fraxinus angustifolia* Vahl.	HCT 116 cells Caco-2 cells	25–100 mg/ml	*In vitro*	Inhibit proliferation, cause apoptosis, increase cleaved PARP-1, caspase 3, and Bax, and decrease Bcl-2 expression	Restivo et al. (2020)
		HT 29 cells	25–100 mg/ml	*In vitro*		
			25–100 mg/ml	*In vitro*		

### Anti-Colorectal-Cancer Effect of Traditional Chinese Medicine Compounds

**FIGURE 1:**
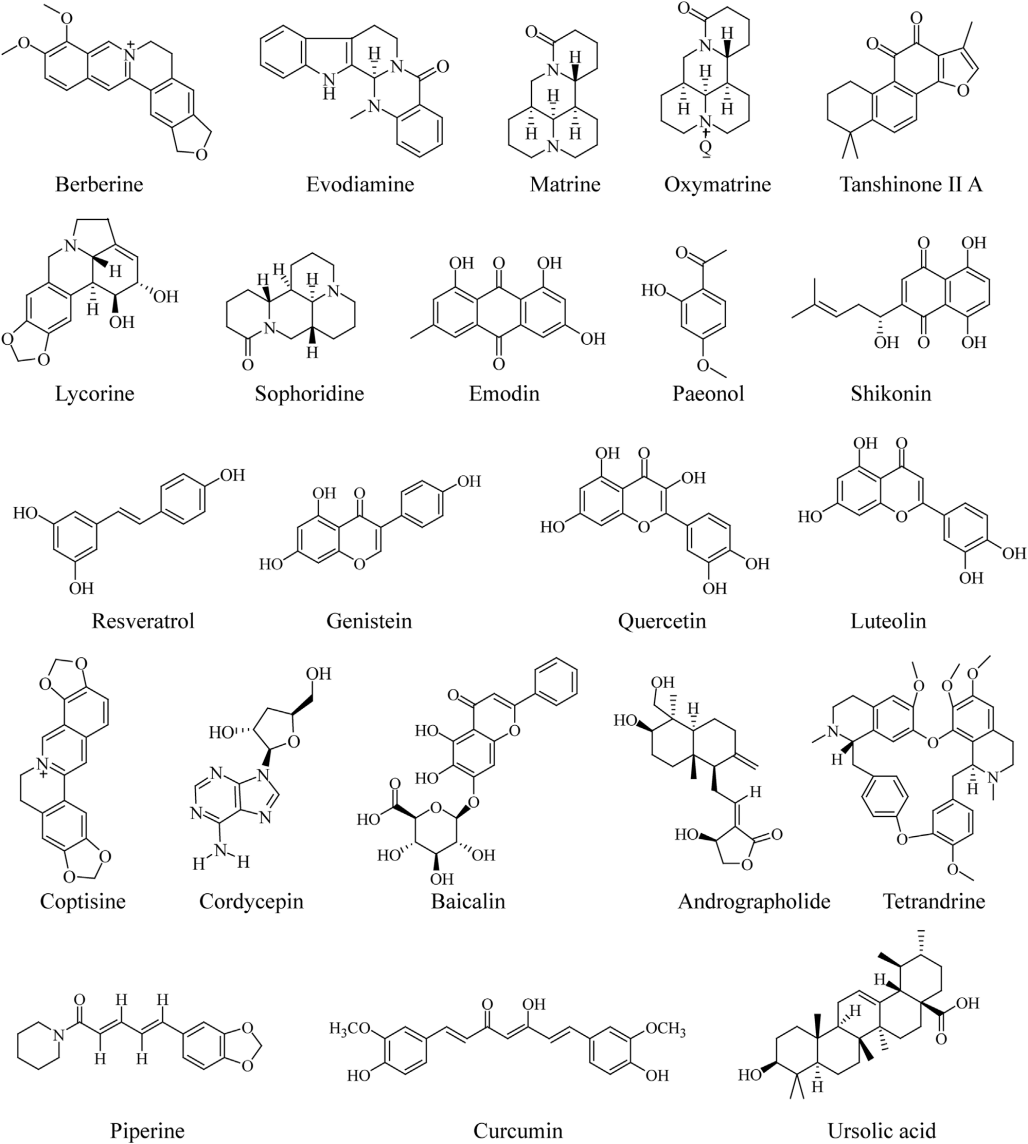
Chemical structures of TCM-derived compounds for CRC.

In recent years, the compounds of TCM have attracted extensive attention in the field of pharmaceutical research, owing to their multiple pharmacological activities and unique advantages of multitarget effect. With the development of modern technology, increasing compounds in TCM have been found to have the effect of anti-CRC (Luo et al., 2019; Kwon and Chan, 2020; Tan et al., 2020). The published reports serve as a jumping-off point for further investigation, with the potential of compounds in TCM to serve as agents for CRC. The chemical structures of the most frequently investigated TCM compounds for CRC are listed in the following (Figure 1).

### Berberine

Berberine, an isoquinoline alkaloid from traditional herbal medicine *Coptis chinensis*, has long been applied as an over-the-counter (OTC) agent for the treatment of intestinal infections and diarrhea (Feng et al., 2019b). With the development of modern pharmacology, the anti-CRC effect of berberine has become increasingly salient (Hallajzadeh et al., 2020). A double-blind, randomized, placebo-controlled clinical trial of berberine in preventing recurrence of CRC corroborated that berberine at 300 mg twice daily was secure and effective in abating the risk of recurrence of colorectal adenoma and might be a recommendable option for chemoprevention after polypectomy (Chen et al., 2020b). Through bioinformatics analysis and validation of related experiments, the researcher proved that berberine exerted the function of inducing G0/G1 phase arrest in HCT116 and SW480 cells by downregulating IGF2BP3 (Zhang et al., 2020c). In another study, treatment of HT-29 and SW480 cells with various concentrations (0.5–20 μM) of berberine resulted in a dose-dependent decrease in sonic hedgehog mRNA and protein (Shen et al., 2020). Moreover, berberine was reported to restrain the expression of GRP78 and its localization on the cell surface in dose- and time-dependent manners, as well as suppress the expression of Bax, Bcl-2, c-Myc and elevate the cytokeratin expression in SW480 cells (Gong et al., 2020). In KM12C cells, berberine can dose dependently attenuate β-catenin function through directly binding to a unique region comprising residues Gln275, Arg316, and Arg371 in nuclear receptor RXRα. Intraperitoneal injection of berberine also hindered the growth of human colon carcinoma xenograft in BALB/c mice with an RXRα-dependent manner (Ruan et al., 2017). In addition, the anti-CRC effect of berberine may be attributed to its promotion of apoptosis (Dai et al., 2019b), regulation of the tumor microenvironment (Yu et al., 2015), and regulation of specific long noncoding RNA (LncRNA) (Dai et al., 2019c). In general, the abovementioned reports suggest that berberine may be a promising therapeutic agent for the treatment of CRC.

### Evodiamine

Evodiamine, a naturally occurring alkaloid with quinazolinocarboline skeleton, originates from numerous herbs including *Evodia rutaecarpa* (Liu et al., 2019b) and *Euonymus europaeus* (Sevastre et al., 2017). In recent years, there have been increasing studies on its anti-CRC effect (Sun et al., 2020b). Evodiamine suppressed the proliferation of CRC cells HT29, HCT15, and SW480 and induced apoptosis by cell cycle arrest in the G_2_/M phase, as well as suppressed the expression of cancer-stem-cell-related genes (Kim et al., 2019a). Moreover, it inhibited metastasis and invasion of CRC through regulating Sirt1-mediated translation in cell and Balb/c mice models (Zhou et al., 2019b). Additionally, evodiamine suppressed the LoVo cells proliferation and promoted apoptosis and lessened HIF-1α either *in vitro* or *in vivo*, as well as decreased the phosphorylation of Akt1/2/3 and the expression of IGF-1 (Huang et al., 2015). In another report, evodiamine was found to be effective in decreasing the expression level of miR-429 in CRC patient tissue (Liu et al., 2016). Furthermore, evodiamine regulated the activity of the p53 signaling pathway to promote the apoptosis of human CRC cells HCT116 and lower the MMP3 expression *via* deactivating the JAK2/STAT3 pathway by the reduction of PGI to suppress migration of cancer cells and decrease the levels of the secreted form of autocrine motility factor (AMF) (Zhao et al., 2015). In combination therapy, evodiamine could inhibit multidrug resistance of HCT116 cells by blocking the p50/p65-NF-kappa-B signaling pathway (Sui et al., 2016). These works provide direct evidence for the anti-CRC effect of evodiamine and also indicate that it may be a potential candidate agent for the treatment of this malignant tumor.

### Curcumin

Curcumin is a kind of acidic polyphenols compound extracted from the rhizomes of *Curcuma longa L.* Growing experimental evidence indicates that curcumin exhibits multitarget biological activities in disease prevention and treatment (Patel et al., 2020). In recent years, curcumin, as a natural anticancer agent (Mortezaee et al., 2019; Tomeh et al., 2019), especially for CRC (Gupta et al., 2019), has captured the attention of pharmaceutical researchers. It was documented that curcumin can abate the viability and proliferation of HCT-116 cells, suppress its migration and invasion to the lung in the mice model, and augment the mRNA and protein levels of apoptosis-related genes (FAS, FADD, caspase-8, and caspase-3) and E-cadherin (Xiang et al., 2020). Furthermore, curcumin suppresses tumor growth in colitis-associated CRC and the proliferation and invasive behavior in CT26 cells by the modulation of the Wnt pathway and E-cadherin. Fortunately, curcumin also exhibited a similar anti-CRC effect on the C57BL/6 mice model (Marjaneh et al., 2018b). Moreover, the use of curcumin accelerated ROS-mediated cell death at 40 μM in the mutated p53 and wild-type p53 colon adenocarcinoma cell lines *in vitro* (Sritharan and Sivalingam, 2021). Moreover, curcumin attenuated tumor EMT by blocking the Wnt signaling pathway and elevating the expression level of naked cuticle homolog 2 (NKD2) in SW620 cells (Zhang et al., 2016). Additionally, curcumin could also exert an anti-CRC effect through epigenetic (Wu et al., 2020) and covalent modification of cysteine 67 residue of SIRT1 (Lee et al., 2018a). Given that resistance of cancer cells to chemotherapeutic agents has been recognized in clinical settings, in recent years, curcumin combined with other chemotherapeutic agents to ameliorate the drug resistance of tumor cells has become paramountly vital. A recent work has provided compelling evidence that curcumin could moderate the expression level of the excision repair cross-complementing gene (ERCC1) and sharply increase oxaliplatin sensitivity in resistant human CRC cells HCT116 through its effects on miR-409-3p (Han et al., 2020). In addition, curcumin could also retard the EMT process by regulating the TET1-NKD-Wnt signaling pathway and then reverse 5-FU resistance of colon cancer cells HCT116 (Lu et al., 2020b), as well as reinforce cisplatin resistance of colon cancer cells HCT 8 by targeting LncRNA-KCNQ1OT1 (Zheng et al., 2021). These effects of curcumin may provide a positive avenue for future research and development of novel agents for CRC.

### Resveratrol

Resveratrol is a kind of natural nonflavonoid polyphenols existing in *Polygonum cuspidatum*, grape, and other herbs. It possesses antioxidant, anti-inflammatory, antishock, and other biological activities and owns a favorable protective effect on the cardiovascular and nervous system (Tian and Liu, 2020). Intriguingly, in the past few decades, its widespread consumption has been reported to work as a cancer preventative approach in several epidemiological studies (Rauf et al., 2018; Singh et al., 2019; Verdura et al., 2020), especially the CRC (Li et al., 2019a). It was reported that resveratrol at 5 µM suppressed TNF-beta-promoted NF-κB-mediated gene biomarkers linked with proliferation, apoptosis, and invasion, as well as lessened TNF-beta/TNF-beta-receptor-mediated inflammatory response in human CRC cells HCT116 *in vitro* (Buhrmann et al., 2019). Another study revealed that there was a statistically significant reduction of cell number and increase in the percentage of apoptosis in CRC cells treated with resveratrol at 1–100 µM (San Hipolito-Luengo et al., 2017). In a study on the effect of resveratrol on CRC metastasis, the authors speculated that resveratrol, with its ability to induce the expression of RKIP at protein levels, may provide a novel option for revealing the structural arrangements during drug–target interactions (Dariya et al., 2020). Furthermore, HCT116 and SNU81 cells treated with various concentrations of resveratrol illustrated inhibition on the proliferation and invasion/metastasis *via* activating tristetraprolin (TPP) (Lee et al., 2018b). The rapid metastasis of CRC, which has a strong link with the invasion degree of cancer cells, can reportedly be undermined by resveratrol through regulating cancer cell invasion *via* the modulation of the levels of focal adhesion molecules (Buhrmann et al., 2017). Resveratrol also induced p53 in CRC through elevating the expression of SET domain containing lysine methyltransferase 7/9 (SET7/9) (Liu et al., 2019a). Also, consistent with its long-standing antioxidant effect, resveratrol has been shown to exhaust the level of thiobarbituric acid reactive substances in blood serum and retard the development of the tumor, in a SD rat model of colon cancer (Rytsyk et al., 2020). In addition to these mechanisms mentioned above, resveratrol could also engender the anti-CRC effect *via* inhibiting cross talk between CRC cells and stromal cells in the multicellular tumor microenvironment (Buhrmann et al., 2020) and reversing the drug resistance of chemotherapy (Huang et al., 2019). As a whole, future studies on the anti-CRC effect of resveratrol will be highly interesting, if these effects can be repeated in other CRC models.

### Quercetin

Quercetin is a natural flavonoid with outstanding biological activity. Research over the past few decades has corroborated that this functional dietary flavone possesses vast pharmacological activities, such as antioxidant, anti-inflammatory, bone protection, cardiovascular protection (Almeida et al., 2018; Huang et al., 2020a; Wong et al., 2020). Interestingly, recent studies have hinted that quercetin may be effective for the prevention and treatment of CRC (Darband et al., 2018; Pang et al., 2019). For example, quercetin inhibited azoxymethane/dextran sulfate sodium- (AOM/DSS-) induced colon carcinogenesis in the C57BL/6J mice model through abating the expression of oxidative stress markers, such as lipid peroxide (LPO), nitric oxide (NO), superoxide dismutase (SOD), glucose-6-phosphate (G6PD), and glutathione (GSH) (Lin et al., 2020). Besides, quercetin alleviated colon damage and lessened the mortality rate in CRC mice, suppressed the TNF-α level, raised the relative abundance of *Parabacteroides*, and decreased the gene of Hmgcs2, Fabp2, and Gpt (Qi et al., 2019). Quercetin also preferentially promotes apoptosis in KRAS-mutant CRC cells *via* JNK signaling pathways (Yang et al., 2019b). Based on the model of BALB/c mice with metastatic CRC, quercetin remarkably hindered lung metastasis of CRC CT26 cells. Also, it blocked the migration and invasion of CT26 cells by regulating the expression of MMPs and tissue inhibitors of metalloproteinases (TIMPs) (Kee et al., 2016). In another study, quercetin inhibited the viability of HT29 cells, caused cell shrinkage, chromatin condensation, and nuclear collapse, lessened the protein expression levels of phosphorylated-Akt, and augmented the protein degradation of constitutive photomorphogenesis 6 signalosome (CSN6) (Yang et al., 2016). Furthermore, quercetin was known to be efficient at facilitating the expression of Slpi (secretory leukoprotease inhibitor) that in turn reduced the inflammatory response *in vivo* (De Santis et al., 2016). Quercetin is a logical botanical ingredient for future treatment of CRC and may be a beneficial addition to the development of other agents for CRC.

### Shikonin

Shikonin is a naphthoquinone compound extracted from traditional Chinese herbal medicine *Lithospermum erythrorhizon* Sieb.et Zucc., which has anti-inflammatory, antioxidation, antitumor, wound healing promoting, and other effects (Guo et al., 2019). In recent years, the antitumor effect of shikonin has been extensively investigated. A multitude of *in vitro* and *in vivo* experiments have indicated that it can effectively curb the occurrence and development of breast cancer, cervical cancer, CRC, and so on (Fan et al., 2012; Tang et al., 2020; Bao et al., 2021). The study on the potential target, molecular mechanism, and antitumor effect of shikonin against CRC *in vivo* and *in vitro* demonstrated that it facilitated human CRC cells apoptosis and autophagy through targeting the galectin-1 and JNK signaling pathway, which delineated a promising and potential novel therapy for CRC (Zhang et al., 2020a). A study regarding the effect of shikonin on the early inflammation model of colon cancer induced by AOM/DSS demonstrated that it could protect the intestinal tissue of animals by hindering the shortening of the colorectum and ulcer formation and abate the expression of cyclooxygenase-2 and inducible nitric oxide synthase, as well as suppress the production of interleukin-6 and activation of nuclear factor-B (Andujar et al., 2018). In another study, the researcher argued that shikonin promoted ROS-based mitochondria-mediated apoptosis in the colon cancer cells SW480 and HCT116. Also, comfortingly, shikonin showed minimal toxicity to nonneoplastic colon cells and no liver injury in BALB/c nude mice xenograft models, suggesting pleasurable safety in the treatment of CRC (Liang et al., 2017). Additionally, the effects of shikonin on the survival and tumor growth of the nude mice model, as well as the migration and invasion of human CRC cells, appear to be able to imply the fact that shikonin inhibited the metastasis of CRC through SIRT2- (silent information regulators 2-) mediated antitumor effect (Zhang et al., 2017b). Furthermore, the anti-CRC activity of shikonin was also validated by the approaches of metabolomics, transcriptomics, and proteomics (Chen et al., 2020c; Chen et al., 2020d). In short, shikonin is a potentially effective agent for the treatment of CRC, which has attention-grabbing clinical application and research value. Although there is increasing research on this active ingredient in the treatment of CRC, the mechanisms of shikonin against CRC are still not fully clear. More detailed information concerning anti-CRC of TCM compounds is depicted in Table 4.

**TABLE 4:** Anti-CRC effect and mechanism of TCM compounds.

Compound name	Main source	Cell lines/model	Dose	Detail	Mechanism	Ref
Berberine	*Coptis chinensis*	HCA 7 cells	10–100 µM	*In vitro*	Result in a downregulation of 33 genes differently involved in cell cycle, differentiation and EMT	Palmieri et al. (2019)
Evodiamine	*Evodia rutaecarpa*	HCT 116 cells	5–15 µM	*In vitro*	Inhibit the proliferation of cells, cause accumulation of cells in S and G2/M phases, and reduce the levels of the secreted form of AMF	Zhao et al. (2015)
Matrine	*Sophora flavescens*	SW 480 cells	0.25–1.25 mM	*In vitro*	Trigger cell apoptosis and G0/G1 cell cycle arrest *via* mediation of microRNA-22	Liu et al. (2020a)
		SW 620 cells	0.25–1.25 mM	*In vitro*		
Oxymatrine	*Sophora flavescens*	RKO cells	0.125–8 mg/ml	*In vitro*	Inhibit the migration of human colorectal carcinoma *via* the inhibition of PAI-1 and the TGF-β1/Smad signaling pathway	Wang et al. (2017)
Coptisine	*Coptis chinensis*	HCT 116 cells	2.81–140.54 µM	*In vitro*	Induce apoptosis of cells by the PI3K/Akt and mitochondrial-related apoptosis pathway	Han et al. (2018)
		BALB/c mice	50–150 mg/kg	*In vivo*		
Lycorine	*Lycoris* plants	RKO cells	10–50 µM	*In vitro*	Induce the activation of the caspase-dependent mitochondrial apoptotic pathway	Wu et al. (2018)
		SW 480 cells	10–50 µM	*In vitro*		
Piperine	*Piper longum* L.	HT 29 cells	1.25 and 2.5 μg/ml	*In vitro*	Enhance radiosensitization by inducing the cells to apoptosis	Shaheer et al. (2020)
Sophoridine	*Sophora alopecuroide*	HCT 116 cells	40–160 µM	*In vitro*	Inhibits human colorectal cancer progression *via* targeting the MAPKAPK2	Wang et al. (2019)
		SW 480 cells	40–160 µM	*In vitro*		
		RKO cells	40–160 µM	*In vitro*		
Tetrandrine	Stephaniae tetrandrae radix	SW620 cells	0.2–50 µM	*In vitro*	Suppress adhesion, migration, and invasion *via* the inhibition of nuclear factor-kappa B, MMP-2, and MMP-9	Juan et al. (2018)
Vinblastine	*Catharanthus roseus*	HCT 116 cells	0.3–2.5 nM	*In vitro*	Inhibit tumor growth and promote angiogenesis factors	Auyeung et al. (2014)
		BALB/c mice	0.25 mg/kg	*In vivo*		
Homoharringtonine	*Cephalotaxus fortunei*	LoVo cells SW480 cells Caco-2 cells	0.1–0.4 µM	*In vitro*	Suppress cell growth by inhibiting EphB4 and the PI3K/AKT and MAPK/EKR1/2 signaling pathways	Shi et al. (2020)
			0.1–0.4 µM	*In vitro*		
			0.1–0.4 µM	*In vitro*		
		BALB/C mice	0.25–1 mg/kg	*In vivo*		
Curcumin	*Curcuma longa* L.	HCT 8 cells	10 µM	*In vitro*	Downregulate KCNQ1OT1 expression, thus reversing cisplatin resistance in CRC cells	Zheng et al. (2021)
		Nude mice	1 g/kg/week	*In vivo*		
Resveratrol	*Veratrum* Linn*.*	DLD-1 cells Caco-2 cells	40–120 µM	*In vitro*	Regulate several genes involved in the modulation of apoptosis such as PMAIP1, BID, and ZMAT3	Gavrilas et al. (2019)
			40–120 µM	*In vitro*		
Quercetin	*Sophora japonica* L.	Wistar rats	50 mg/kg	*In vivo*	Suppress DNA damage and induce DNA repair and increase the levels and activities of enzymic, as well as the nonenzymic antioxidants	Darband et al. (2020)
Tanshinone II A	*Salvia miltiorrhiza* Bge.	SW 620 cells	0.5–10 μg/ml	*In vitro*	Suppress SW620 proliferation and induce apoptosis	Xue et al. (2019)
Luteolin	*Reseda odorata*	HT-29 cells SW480 cells SW620 cells LoVo cells	10–100 µM	*In vitro*	Upregulate miR-384 and downregulate the PTN expression level both in CRC cells and tissues	Yao et al. (2019)
			10–100 µM	*In vitro*		
			10–100 µM	*In vitro*		
			10–100 µM	*In vitro*		
		BALB/c mice	100 mg/kg	*In vivo*		
Genistein	Puerariae lobatae Radix	SW480 cells	25–100 µM	*In vitro*	Increase the expression of TGF-β1 and lncRNA TTTY18, followed by upregulated Ki-67, serum, and SGK1	Chen et al. (2020a)
Baicalin	*Scutellariae* Radix	RKO cells	50, 100 μg/ml	*In vitro*	Inhibit cell growth, migration, and invasion and induce cell apoptosis, induce cell cycle arrest in the G1 phase, and suppress both endogenous and exogenous TGFβ1-induced EMT	Yang et al. (2020)
		HCT 116 cells	50, 100 μg/ml	*In vitro*		
		BALB/c mice	100, 200 mg/kg	*In vivo*		
Shikonin	*Arnebiae* Radix	SW 480 cells	2.5–15 µM	*In vitro*	Induce mitochondria-mediated apoptosis by Bcl-2 family protein and increase the intracellular ROS	Liang et al. (2017)
		HCT 116 cells	2.5–15 µM	*In vitro*		
		BALB/c mice	3, 6 mg/kg	*In vivo*		
Emodin	Rhei Radix et Rhizoma	HCT 116 cells	15–60 μg/ml	*In vitro*	Block the growth and invasion of CRC cells by restraining VEGFR2	Dai et al. (2019a)
		BALB/c mice	20–80 mg/kg	*In vivo*		
Cordycepin	*Cordyceps sinensis*	HCT 116 cells	62.5–540 µM	*In vitro*	Inhibit cell growth by the endogenous Bax-dependent mitochondrial apoptosis pathway	Li et al. (2019b)
Paeoniflorin	*Paeonia lactiflora* Pall.	HCT 116 cells	2.5–40 mM	*In vitro*	Inhibit migration and invasion and suppress cell metastatic potential and decrease the expression of HDAC2 and vimentin, increasing E-cadherin	Zhang et al. (2018)
		SW 480 cells	2.5–40 mM	*In vitro*		
		BALB/c mice	1 g/kg	*In vivo*		
Ginsenoside Rh3	Ginseng Radix et Rhizoma	SW 1116 cells	60–240 μg/ml	*In vitro*	Inhibit proliferation and increase the ratio of apoptotic cells, mRNA, and protein of caspase3	Cong et al. (2020)
		BALB/c mice	100, 200 mg/kg	*In vivo*		
Andrographolide	*Andrographis* Herba	HCT 116 cells	5–100 µM	*In vitro*	Anti-TNF-α-induced IL-8 by inhibition of NADPH oxidase/ROS/NF-κB and Src/MAPKs/AP-1 signaling pathways	Yuan et al. (2018)
Ursolic acid	*Ligustri lucidi* Fructus	HCT 116 cells HCT 8 cells	10–40 µM	*In vitro*	Suppress the invasive by regulating the TGF-β1/ZEB1/miR-200c signaling pathway	Zhang et al. (2019a)
			10–40 µM	*In vitro*		
Celastrol	*Tripterygium wilfordii*	HCT 116 cells	2.5–10 µM	*In vitro*	Inhibit proliferation, migration, and NOS activity in the cytoplasm and inhibit growth and migration	Gao et al. (2019)
		HT 29 cells	2.5–10 µM	*In vitro*		
Bufalin	*Bufonis venenum*	HCT 116 cells	0.1–50 µM	*In vitro*	Reverse acquired drug resistance by inhibiting stemness in colorectal cancer cells	Sun et al. (2017)
		LoVo cells	0.1–50 µM	*In vitro*		
		BALB/c mice	1 mg/kg	*In vivo*		
Norcantharidin	*Mylabris phalerata* Pallas.	HT 29 cells	5, 10 μg/ml	*In vitro*	Cause proapoptotic and antiglycolytic effects through modulation of Fam46c expression and inhibition of ERK1/2 signaling	Zhang et al. (2020b)
			5, 10 μg/ml	*In vitro*		
		LoVo cells SW 620 cells	5, 10 μg/ml	*In vitro*		
Scutellarin	*Erigerontis* Herba	HCT 116 cells	20–100 µM	*In vitro*	Reduce viability and induce apoptosis, reduce Bcl-2, and increase Bax and phosphorylation of p53	Yang et al. (2017)
Paeonol	*Salvia miltiorrhiza* Bge.	HCT 116 cells	7.8125–500 μg/ml	*In vitro*	Induce G_0_/G_1_ phase arrest and cell apoptosis by inhibiting the Wnt/β-catenin signaling pathway	Liu et al. (2020b)

**FIGURE 2:**
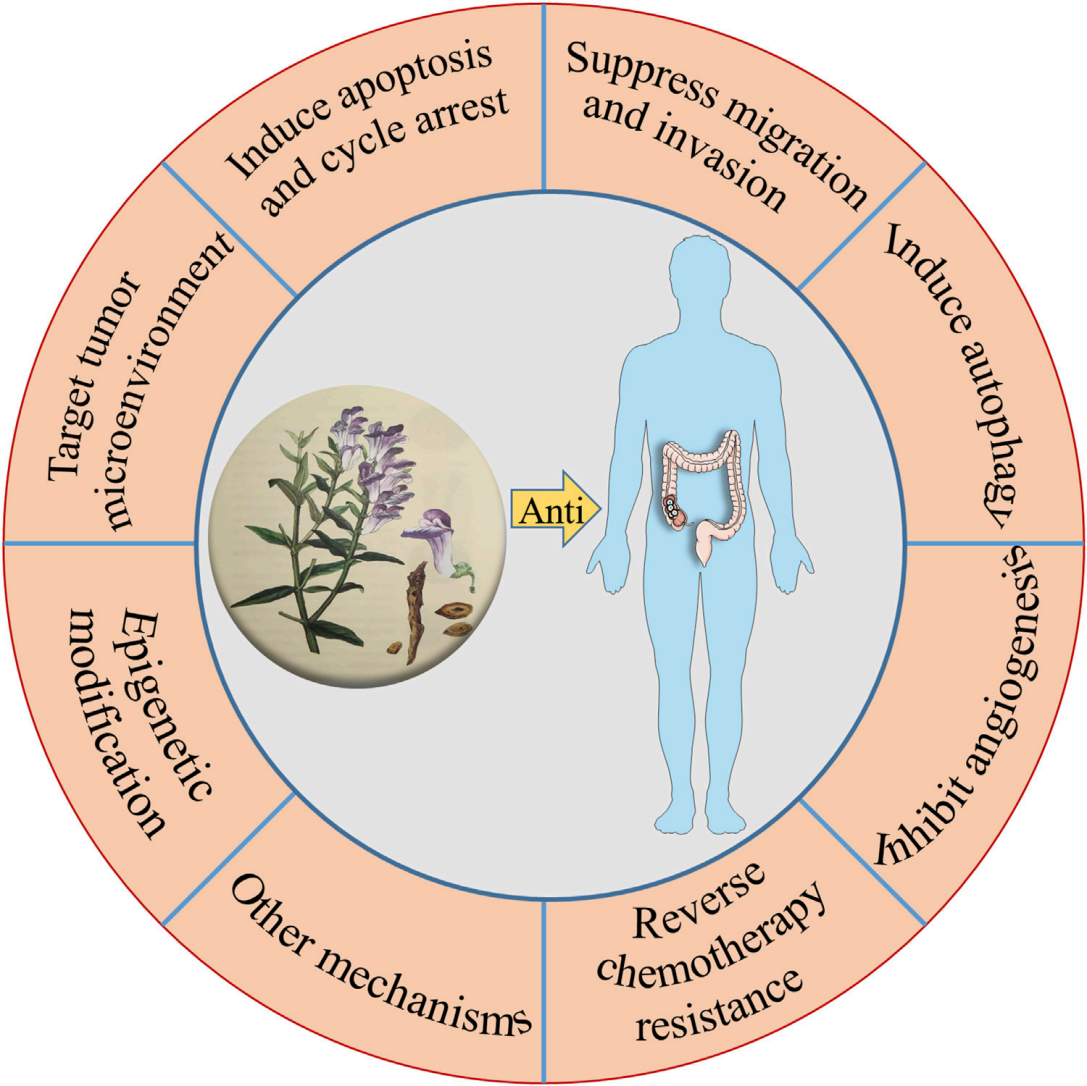
The main mechanisms of TCM for the treatment of CRC.

To sum up, the compounds in TCM have shown great potential in anti-CRC. In terms of mechanism, as can be seen throughout this review, it is easy to realize that these anti-CRC activities mentioned above are related to the inhibition on the proliferation, migration, induction of apoptosis, and autophagy (Chen et al., 2021), as well as the target of the tumor microenvironment (Figure 2). The research studies on the anti-CRC effects of TCM listed above are only some instances. Consequently, this is a wide-open field for additional investigation, with other novel components in TCM possessing anti-CRC activity needed to be explored. In the future, these Chinese herbal medicinal ingredients and biochemical agents may orchestrate the anti-CRC effects in clinical practice.

## Discussion and Perspectives

With a unique philosophy and clinical treatment principles, TCM strictly relies on the two vital therapeutic methods of holism and syndrome differentiation. A rich clinical experience has accumulated in the treatment of multiple diseases. Although some reports, including clinical trials, have demonstrated that TCM formulations have advantages against multiple targets in the treatment of multiple diseases, it still has some limitations. First of all, TCM is rich in a considerable number of chemical components, and each component produces a variety of biological effects through interaction. Therefore, it is difficult to clarify the relatively clear material basis, target, and molecular biological mechanism. Moreover, it is worthy of further explanation whether the numerous targets of TCM ingredients also mediate its adverse reactions. Second, increasing studies showed that most of the components in TCM, such as alkaloids, flavonoids, and saponins, have some defects, such as low solubility, needy bioavailability, and poor stability, which undoubtedly limited the further application and promotion of TCM products to some extent. In addition, the current research of TCM mainly focuses on one or several single components, which is divorced from the guidance of TCM theory. Whether these single active ingredients can replace the effective material basis of the whole TCM is worth thinking deeply. Besides, TCM is also faced with the problems of pharmacokinetic/pharmacodynamic (PK/PD) uncorrelation in the evaluation of drug formation. Consequently, it is urgent to excavate more technologies and means for the discovery of TCM.

### Excavating the Effective Material Basis of Anti-Colorectal-Cancer Traditional Chinese Medicine Based on Multiple Types of Target Omics Analysis

The effective substance of TCM refers to the chemical composition (group) in TCM or CMF, which can intuitively express the clinical efficacy of pharmaceutical ingredients. Currently, although there are extensive works on the anti-CRC effect of TCM, the effective substance remains elusive. In addition, TCM possesses the characteristics of multicomponent, multitarget, and synergistic effects with a relatively complex metabolic process *in vivo*. Also, the phenomenon of synergy or inhibition between components in the process of absorption and metabolism frequently occurs. In recent years, owing to the development of novel technologies, a series of multitypes of omics analysis, such as transcriptomics, metabolomics, and proteomics, have entered the field of the discovery of effective substances of TCM one after another, which will contribute to the lucidity of the material basis of TCM in anti-CRC. In the future, we can systematically characterize the efficacy material base of TCM against CRC through multiomics analysis and comprehensively elaborate the procedure of ingredient intervention in cancer employing genomics, metabolomics, proteomics, and bioinformatics, so as to more systematically explicate the connotation of the material basis of TCM against CRC. These research studies are capable of resolving the decipher relationship of “Prescription–Disease–Syndrome” to a certain extent and organically combining “molecular identification of disease and syndrome-pharmacodynamic material basis-key molecular mechanism verification” to assist the development of “precision medicine” of TCM against CRC.

### Guiding the Development and Application of Anti-Colorectal-Cancer Traditional Chinese Medicine by Reverse Pharmacology

**FIGURE 3:**
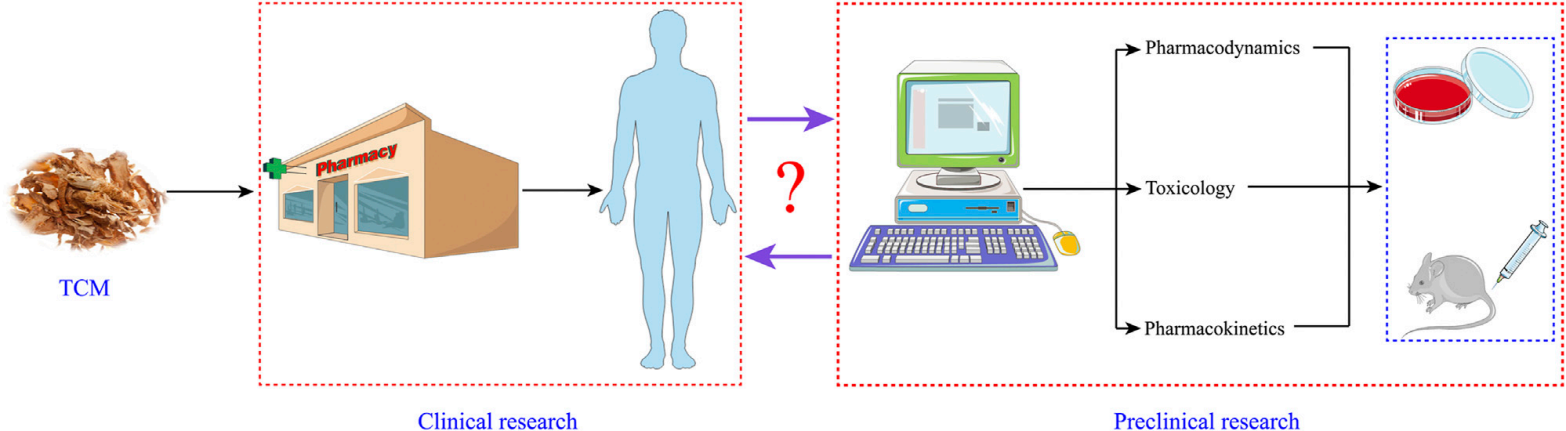
Reverse pharmacology guiding the development and application of anti-CRC Chinese medicine.

The development of modern chemical agents mostly adopts the research procedure of “Bench (preclinical study)-to-Bedside (clinical practice).” However, the vast majority of TCM has entered clinical practice under the guidance of TCM theory without undergoing the “Bench.” It means that TCM or medicine material crude slices can render the patients directly through the pharmacy management channel or pharmacy of the hospital without relevant pharmacological studies. Although TCM practitioners own affluent experience in this approach of medication, preclinical studies such as pharmacodynamics, pharmacokinetics, and toxicology ought not to be ignored. Hence, there is a gap with regard to its safety and effectiveness, namely, sufficient and credible preclinical evidences and systematic randomized clinical trials. Taking the clinical TCM for the treatment of CRC as an example, although this ancient theory claims that TCM has a positive effect in the treatment of this malignant tumor, the pharmacokinetic parameters, potential targeting mechanism, and toxicological information of different components in it have not been entirely expounded. Therefore, we must unearth more preclinical research data to guide the clinical application of anti-CRC TCM. In a sense, this is the process of reverse pharmacology guiding the development and application of anti-CRC Chinese medicine (Figure 3).

### Scientific and Reasonable Nanostrategy to Make Up for the Deficiency of Clinical Transformation of Anti-Colorectal-Cancer Traditional Chinese Medicine

**FIGURE 4:**
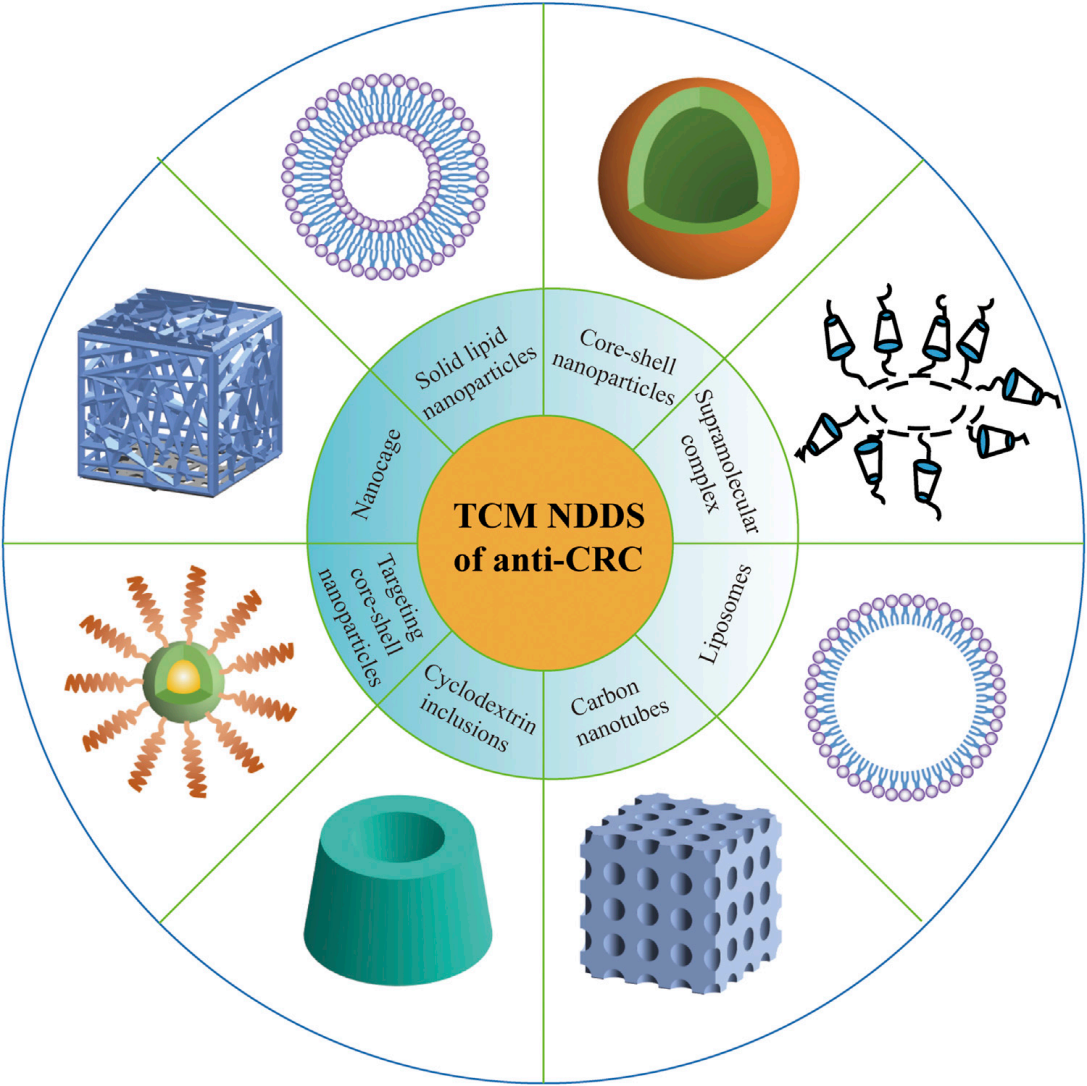
Nanodelivery system of TCM for the treatment of CRC.

The active ingredients of anti-CRC on the market or in the development stage currently mostly originate from natural Chinese herbal medicines, which have promising antitumor effects and less toxic and side effects, as well as enhancement of human immunity. Nevertheless, most of the antitumor active ingredients of Chinese herbal medicine show some issues, such as poor aqueous solubility (Rafiee et al., 2019), needy bioavailability (Ban et al., 2020), and awful specific distribution (Huo et al., 2020). In recent years, although the applications of nanotechnologies such as liposome, nanoparticles, polymer micelles, microemulsion, and the biomimetic drug delivery system have eliminated the abovementioned flaws of active ingredients in TCM, there are still some problems that do exist. For example, Japanese researchers applied albumin combined with paclitaxel nanoparticles to carry out a phase III clinical study on 741 patients with advanced gastric cancer who were not sensitive to first-line chemotherapy. The results demonstrated that the median survival time of patients treated with nano-paclitaxel was only slightly better than that of patients treated with conventional paclitaxel (Shitara et al., 2017). In addition, a project of 1,206 patients with early breast cancer also showed that although albumin combined with paclitaxel nanoparticles performed significantly better than conventional paclitaxel preparations in inhibiting tumor metastasis, there was no significant difference in long-term survival between the two treatment groups (Untch et al., 2019). These studies suggest that *in vitro* cell models and *in vivo* animal models often do not truly reflect the pathophysiological process of the human body, which may also be because the research on the correlation between them relatively remains scarce. Besides, it is well known that the effective components of TCM against CRC own the effect on multitarget. Like that, does this seemingly advantageous feature also result in the situation that the targeting effect of TCM components too scattered? Therefore, although the effective components of TCM against CRC can be nanomodified, it is essential to establish a scientific, reasonable, and systematic evaluation system (Figure 4). Also importantly, future works may reap huge fruits from having components of TCM against the CRC structure modified, and this will be conducive to formulate corresponding strategies to overcome the shortage of clinical transformation of anti-CRC agents.

### Detecting the Anti-Colorectal-Cancer Mechanism of Effective Components of Traditional Chinese Medicine Based on the Whole Effect Mediated by Gut Microbiota

The study of drug metabolism *in vivo* has always been a paramount link in the field of pharmacy and the development of novel agents, as the favorable oral absorption properties and excellent bioavailability are the current international indicators to evaluate the drug properties. In recent years, the efficacy of several active ingredients of TCM against CRC has been confirmed in clinical practice. However, there is a special phenomenon in the field of TCM that should be taken into account. A considerable number of active ingredients exert therapeutic effects or even intense influence, but with a low exposure in the blood. Most of these components stem from natural products with various structures (such as alkaloids, flavonoids, terpenoids, and saponins), which generally possess the characteristics of a definite curative effect, but unfavorable intestinal absorption and unsatisfactory oral bioavailability. Therefore, these characteristics give rise to the re-exploration of the existing concept of drug bioavailability, namely, whether the drug molecule itself as the only indicator of bioavailability is correct or not. Lately, gut microbiota as the “Forgotten and Invisible Organ” of the human body has been extensively concerned. The latest research harbored the idea that the gut microbiota is closely related to the occurrence and development of CRC (Vivarelli et al., 2019; Song et al., 2020). For the metabolism or transformation of active ingredients in oral Chinese medicine, gut microbiota is still an unknown territory, as the type and quantity of biochemical enzymes in the intestinal cavity may be more than those in other organs in the body; thus, the products or degradation fragments produced after metabolism may be more complicated (Zhao et al., 2021). Thus, it is of great significance to probe the gut microbiota for the metabolism or transformation of oral insoluble TCM (Feng et al., 2019a; Sun et al., 2020a). Berberine can stimulate intestinal microorganisms to produce metabolites with rosy safety and biological activity, such as short-chain fatty acids (SCFAs). When studying the anti-CRC effect of natural ingredients, we should take the prototype agents and the endogenous metabolites produced by gut microbiota as a whole based on the interaction between drugs and gut microbiota to expound the pharmacokinetics and pharmacodynamic mechanism *in vivo*, which may also be a valuable implication for the treatment of chronic tumors including CRC in the future.

## Conclusion

Over the past few decades, the incidence rate of CRC in most countries or regions of the world has been remarkably increased, which undoubtedly increases the health burden of various countries on the treatment of this malignant malady. The existing chemotherapy approaches for CRC possess evident adverse reactions and dreadful complications; thus, it is urgent to develop novel specific drugs to curb this unfavorable situation. Owing to the increasing R&D time and cost of chemosynthetic agents, the majority of the pharmaceutical developers pay increasing attention to unearthing novel and efficient natural ingredients and their derivatives from TCM for the treatment of CRC. However, due to the existence of adverse factors such as the difference of administration methods and the complexity of clinical trial conditions, the efficacy and mechanism of the most effective components of TCM in the treatment of CRC are inconclusive. Therefore, there is an urgent need for large-scale multicenter randomized controlled trials for TCM against CRC.

In view of that animal models often do not truly reflect the pathophysiological process of the human body, there is still a long way to go for nanodrugs to be popularized/transformed from animal-level simulation to human clinical application, where the effectiveness of nanodrugs *in vivo* has been queried. Of course, it is more convincing that the effectiveness of nanodrugs can be proved in the human body, but the current conditions are arduous to achieve. In order to better evaluate the process and antitumor effect, models more similar to the human physiological environment need to be further explored. At present, the tumor-bearing mouse model relatively truly reflects the physiological environment of the human body to a certain extent. Although this is not the best evaluation method, it is also adequate to expound the problem, which also lays a solid foundation for human experiments in the future. In addition, strengthening the correlation between the preclinical research model and the fate of nanocarriers in the human body will also contribute to overcome this limitation.

Clinical trials require to be initiated to more accurately test and verify whether TCM is effective in the treatment of CRC. In order to accelerate the clinical transformation of TCM products, numerous factors need to be taken into account, such as the drug dose variance, interindividual patient variability, and differences between animal models and patients, which make the design of clinical trials full of challenges. In addition, at present, the research of TCM on CRC mostly dwells in the molecular level. With the emergence of novel technologies such as whole-genome sequencing, genome editing, and quantitative proteomics analysis, we can have a more thorough and accurate understanding of the molecular targets and signal pathways regarding its anti-CRC effect. As investigation into this field continues, hopefully, the intervention of anti-CRC TCM can be combined with other therapies currently being developed, to apply these ancient herbal medicines in a more safe and reasonable way based on conclusive evidence.
